# UCP-2 and UCP-3 Proteins Are Differentially Regulated in Pancreatic Beta-Cells

**DOI:** 10.1371/journal.pone.0001397

**Published:** 2008-01-02

**Authors:** Yunfeng Li, Kathrin Maedler, Luan Shu, Leena Haataja

**Affiliations:** Larry L. Hillblom Islet Research Center, The David Geffen School of Medicine, University of California at Los Angeles, Los Angeles, California, United States of America; Mayo Clinic College of Medicine, United States of America

## Abstract

**Background:**

Increased uncoupling protein-2 (UCP-2) expression has been associated with impaired insulin secretion, whereas UCP-3 protein levels are decreased in the skeleton muscle of type-2 diabetic subjects. In the present studies we hypothesize an opposing effect of glucose on the regulation of UCP-2 and UCP-3 in pancreatic islets.

**Methodology:**

Dominant negative UCP-2 and wild type UCP-3 adenoviruses were generated, and insulin release by transduced human islets was measured. UCP-2 and UCP-3 mRNA levels were determined using quantitative PCR. UCP-2 and UCP-3 protein expression was investigated in human islets cultured in the presence of different glucose concentrations. Human pancreatic sections were analyzed for subcellular localization of UCP-3 using immunohistochemistry.

**Principal Findings:**

Dominant negative UCP-2 expression in human islets increased insulin secretion compared to control islets (*p*<0.05). UCP-3 mRNA is expressed in human islets, but the relative abundance of UCP-2 mRNA was 8.1-fold higher (*p*<0.05). Immunohistochemical analysis confirmed co-localization of UCP-3 protein with mitochondria in human beta-cells. UCP-2 protein expression in human islets was increased ∼2-fold after high glucose exposure, whereas UCP-3 protein expression was decreased by ∼40% (*p*<0.05). UCP-3 overexpression improved glucose-stimulated insulin secretion.

**Conclusions:**

UCP-2 and UCP-3 may have distinct roles in regulating beta-cell function. Increased expression of UCP-2 and decreased expression of UCP-3 in humans with chronic hyperglycemia may contribute to impaired glucose-stimulated insulin secretion. These data imply that mechanisms that suppress UCP-2 or mechanisms that increase UCP-3 expression and/or function are potential therapeutic targets to offset defects of insulin secretion in humans with type-2 diabetes.

## Introduction

Prolonged exposure of islets to high glucose or free fatty acid concentrations causes attenuated glucose-stimulated insulin secretion (GSIS) [1], [2]. A number of explanations have been proposed, including decreased proinsulin biosynthesis, desensitization to the glucose signal, depleted insulin stores, chronic oxidative stress and increased beta-cell apoptosis [2]–[4]. Furthermore, chronic high fatty acid concentrations may be beta-cell toxic in the context of concomitant hyperglycemia, referred to as glucolipotoxicity [1], [2], [5]. Glucose oxidation generates NADH and FADH, which donate electrons to the mitochondrial electron transport chain. Protons subsequently re-enter the mitochondrial matrix via ATP synthase to generate ATP. The increase in ATP results in the closure of the ATP sensitive potassium channels, which in turn leads to depolarization of the cell membrane and an influx of ionized calcium thereby triggering insulin secretion [6], [7]. Therefore, an attenuated increment in mitochondrial membrane proton gradient following glucose oxidation would be expected to attenuate the consequent increment in insulin secretion. One mechanism that may cause a decrease in the mitochondrial membrane proton gradient is leakage of protons down this gradient via uncoupling proteins. To the extent that protons re-enter beta-cell mitochondria via uncoupling proteins and release energy in the form of heat, they will be unavailable for ATP synthase to generate ATP [8].

Five different uncoupling protein isoforms are known and they have been identified in different tissues. UCP-1 is expressed predominantly in brown adipose tissue, and it is responsible for shivering thermogenesis in newborn humans and rodents [9], [10]. UCP-2 is ubiquitously expressed, whereas UCP-3 is expressed preferentially in skeletal muscle, brown adipose tissue and minimally in heart [11], [12]. Both UCP-2 and UCP-3 prevent the reactive oxygen species formation and therefore protect living cells against oxidative stress [13], [14]. Furthermore, UCP-3 plays a physiological role in fatty acid metabolism by exporting fatty acids from the mitochondria [15]. UCP-4 and UCP-5 are expressed mainly in the nervous system [16].

UCP-2 expression is increased by exposure of rodent islets to high levels of glucose and free fatty acids [17]–[21]. Furthermore, UCP-2 expression is upregulated in islets of wild type *ob/ob* mice and rats fed a high-fat diet [22]–[24]. When *ob/ob* mice lack a functional UCP-2 gene or when UCP-2 deficient mice were fed with a high-fat diet, GSIS was improved when compared with wild-type mice [22], [25], [26]. Finally, hyperglycemia-induced mitochondrial superoxide activates UCP-2, which subsequently impairs GSIS [21]. These data imply that UCP-2 (and potentially other uncoupling proteins) may be important in the regulation of insulin secretion in health, and may contribute to impaired GSIS in diabetes. To date there is minimal data available in human islets. UCP-2 mRNA transcription is increased by high glucose in human islets [27]. Furthermore, a recent study demonstrated that UCP-2 protein levels are increased by 24% in human islets isolated from diabetic subjects compared to control subjects [28]. The purpose of the present study was to address the following questions: (1) Is UCP-2 protein expression in human islets increased by chronic exposure to high concentrations of glucose, and/or oleic acid? (2) Does endogenous UCP-2 expression regulate GSIS in human islets? (3) Is UCP-3 expressed in human islets, and if so, (4) Does high glucose regulate UCP-3 protein expression? (5) Does UCP-3 over expression regulate GSIS in human islets?

## Methods

### Procurement of human islets and cell culture

Islets from the pancreas of organ donors were obtained through the Juvenile Diabetes Research Foundation human islet distribution program. Islets were cultured at RPMI medium containing 10% heat-inactivated FCS, 2 mM Glutamax and 100 U/ml Penicillin/Streptomycin (described hereafter as complete medium) supplemented with 5.5 mM glucose in humidified air containing 5% CO_2_. All tissue culture reagents were from Invitrogen (Carlsbad, CA).

### Generation of human UCP-2 dominant negative adenovirus

Total RNA was extracted from human islets using a Qiagen RNeasy kit (Qiagen, Santa Clarita, CA), and Omniscript Reverse Transcriptase (Qiagen) was used for each RT reaction. PCR was carried out using Platinium Taq DNA polymerase (Invitrogen) and UCP-2 primers. The PCR primer sequences for human UCP-2 were 5′ATGGTTGGGTTCAAGGCCACAG3′ and 5′TCAGAAGGGAGCCTCTCGGGAAG3′. PCR reactions were performed with 2 min initial denaturing step, followed by 35 cycles of 30 sec at 94°C, 30 sec at 55°C and 1 min at 72°C. PCR samples were run on a 1.2% agarose gel, and inserted into pGEM-T Easy vector (Promega, Madison, WI). The sequencing of PCR fragment confirmed 100% homology with published human UCP-2 sequence (GenBank Accession number: A94592). As well as generating a construct with UCP-2 we also generated a dominant negative UCP-2 construct in order to allow us to establish if the endogenous UCP-2 protein is active in regulation of insulin secretion. The rationale here is that as wild type UCP-2 in its functional form is a dimer, the dominant negative approach allows examination of a reduction in functional wild type protein [29]. To generate the dominant negative (dn) mutation, aspartic acid at position 212 was substituted with asparagine using PCR mutagenesis. This mutation was originally characterized by Mills et al., who demonstrated that the expression of UCP-2_D212N_ raises mitochondrial membrane potential [29]. The entire coding region of dnUCP-2 was inserted in the pAdTrack-CMV expression vector [30]. Ad-dnUCP-2 adenovirus was generated using standard procedures [31]. This adenovirus expresses both dnUCP-2 and green fluorescent protein (GFP). Ad-GFP was kindly provided by Dr. C. Rhodes (University of Chigaco, IL)[32].

### Transduction of humans islets with dnUCP-2 adenovirus

One hundred islets were transduced with adenovirus expressing GFP (Ad-GFP) as a control or adenovirus expressing dominant negative human UCP-2 (Ad-dnUCP-2) at 3×10^6^ pfu/islet. Islets were incubated in viral solution at 37°C. After 3 hr, islets were washed twice in complete medium, and transferred on a 35-mm suspension culture dish.

### Mitochondrial membrane potential detection

Mitochondrial membrane potential was detected by staining living islets with 5,5′,6,6′-tetrachloro-1,1′,3,3′-tetraethylbenzimidazolylcarbocyanine iodide (JC-1, Molecular Probes, Eugene, OR). JC-1 is a lipophilic dye, which selectively enters into the mitochondria and forms red aggregates as the mitochondria membrane becomes more polarized [33], [34]. Islets were cultured on extracellular matrix-coated chamber slides derived from human bladder carcinoma cells (HTB9, [35]), allowing islets to detach to the slide and spread. Three days later, islets were transduced with Ad-GFP or Ad-dn-UCP-2 at 3×10^6^ pfu/islet as described above. Forty-four hours after transduction, medium was replaced with Krebs Ringer buffer (119 mM NaCl, 4.7 mM KCl, 2.5 mM CaCl_2_, 1.2 mM MgCl_2_, 1.2 mM KH_2_PO_4_, 25 mM NaHCO_3_, 2 mM glucose). Islets were stained with 10 µg/ml JC-1 for 30 minutes at 37°C and examined with a confocal microscope (Leica TCS-SP, Leica Microsystems). Total islet area and mitochondia (red) were measured using Image-Pro Plus 4.1 (Media Cybernetics, Silver Spring, MD).

### Insulin release by transduced human islets

Fifteen transduced islets were placed in perifusion chambers and perifused for 40 min with oxygenated Kreb's Ringer bicarbonate buffer, pH 7.4, at basal (4 mM) glucose followed by increased (16 mM) glucose concentration for 60 min as previously described in detail [36], [37]. Briefly, perfusate was collected at one-minute intervals and insulin was measured in duplicate every 4 minutes from 24 to 64 minutes with a two-site insulin immunospecific ELISA. Four separate human islet donors were used. One experiment was performed in duplicate (two separate perfusions per islet batch) and three others in triplicate.

### Generation of human UCP-3 adenovirus

The PCR primer sequences for human UCP-3 were 5′ ATGGTTGGACTGAAGCCTTCAG3′ and 5′TCAAAACGGTGATTCCCGTAAC3′. The sequencing of PCR fragment confirmed 100% homology with published human UCP-3 sequence (GenBank Accession number: U94592). Primers were used to generate *Eco*RI and *Xho*I sites, the entire coding region of UCP-3 was inserted into pENTR2B (Invitrogen, Carlsbad, CA) and subsequently into pAd/CMV/DEST adenovirus vector (Invitrogen). Recombinant adenoviruses expressing human UCP-3 and luciferase (control) were generated and purified according to manufacturer's instructions (Invitrogen).

### Western blot analysis

Total protein samples were prepared in Laemmli sample buffer from human islets. Twenty µg of protein was separated on a 12% SDS-PAGE and transferred to PVDF membranes (Bio-Rad, Hercules, CA). Membranes were blocked with 5% nonfat dry milk in 0.1% Tween/Tris-buffered saline (TBS-T), and incubated overnight at 4°C in 2.5% milk in TBS-T containing polyclonal rabbit anti-UCP-2 (1∶1000; RDI-UCP2MabrX, Research Diagnostics, Flanders, NJ) or rabbit anti-UCP-3 (1∶500; AB-3046, Chemicon, Temecula, CA). The specificity of this UCP-2 antibody has been previously documented [38]. To confirm the specificity of UCP-2 antibody, it was incubated with an excess of UCP-2 control peptide (10 µg of peptide per 1 µg of antibody; RDI-UCP2M-CP, Research Diagnostics) for 4 hr before continuing with the membrane overnight at 4°C. No UCP-2 signal was detected after this incubation. Furthermore, we used a mouse tissue panel to confirm the antibody specificity. This analysis demonstrated that UCP-2 is expressed in mouse spleen and lung, but not in skeletal muscle, as has been shown previously [39]. We chose to use UCP-3 antibody from Chemicon, because this antibody has been previously extensively validated using knock-out mice and adenoviral overexpression [40]–[42]. Membranes were washed five times with TBS-T and incubated with horseradish peroxidase-conjugated goat anti-rabbit IgG (1∶3000; Zymed Laboratories, S. San Francisco, CA) for 1 hr. Proteins were visualized using enhanced chemiluminescence (ECL; Amersham, UK). Filters were stripped using Restore Western Blot Stripping buffer (Pierce, Rockford, IL) and reprobed with anti-β-actin antibodies (1∶1000, Cell Signaling Technology, Beverly, MA). Protein expression levels were analyzed using the Un-Scan-It program (Silk Scientific, Orem, UT).

### Real-time quantitative PCR

Real-time quantitative PCR analysis was performed on LightCycler system (Roche Applied Science, Indianapolis, IN) by SYBR Green I dye detection. The reactions were assembled following the manufacturer's recommendation. Briefly, 20 µl of reaction mixture contain 1x buffer (LC FastStart DNA Master Plus SYBR green, Roche), 1 µM forward and reverse primers, and the human islet cDNA template. The PCR conditions were 10 min at 95°C, followed by 45 cycles of 2 s at 95°C, 2 s at annealing temperature, and 12 s at 72°C. We verified the specificity of PCR by measuring the melting curve of the PCR product at the end of reaction. Fluorescent data are specified for collection during primer extension. The relative cDNA ratio was calculated using α-tubulin as a reference calibrator to normalize equal loading of template cDNA: 5′AGAGTCGCGCTGTAAGAAGC3′ and 5′TGGTCTTGTCACTTGGCATC3′. The PCR primer sequences for human UCP-2 were 5′TCAATGCCTACAAGACC3′ and 5′TCTTGACCACGTCTACA3′, and for human UCP-3 5′TGAAGGTCCGATTTCAG3′ and 5′GATGTCGTAGGTCACCA3′.

### Immunohistochemistry

Human pancreatic specimens of non-diabetic subjects were obtained from normal pancreas removed at surgery for pancreatic adenomas from the Mayo Clinic (Rochester, MN). A pancreatic specimen that morphologically appeared to be unaffected by the underlying disease was fixed in formaldehyde and embedded in paraffin for subsequent staining. Four µM sections were were deparaffinized in toluene, rehydrated in grades of alcohol, and washed in H_2_O. All slides were subject to antigen-retrieval protocols using antigen unmasking buffer (Vector Laboratories, Burlingame, CA). After antigen unmasking, the slides were cooled to room temperature, permeabilized in 0.4% Triton X-100/TBS, and blocked with 0.2% Tween 20/3% IgG-free bovine serum albumin (BSA)/TBS. Primary Ab's were diluted in the blocking solution at the following dilutions: rabbit anti-UCP3, 1∶75 (Chemicon), mouse anti-mitochondria, 1∶200 (ab3298, abcam, Cambridge, MA) and guinea pig anti–insulin 1∶500 (Dako, Carpinteria, CA). Goat-derived secondary Ab's conjugated to FITC or Cy3 were diluted 1∶200 (Jackson ImmunoResearch Laboratories, West Grove, PA). To determine the specificity of UCP-3 antibody, it was incubated with 10-fold excess of UCP-3 control peptide (AG769, Chemicon) for 1 hr before continuing with immunostaining. All slides were mounted with Vectashield (Vector Laboratories). Fluorescent slides were viewed using a Leica DXMRA confocal microscope and images acquired using Volocity software (Improvision, Lexington, MA).

### Glucose-stimulated insulin secretion (GSIS)

For acute insulin release in response to glucose, islets were washed and preincubated (30 min) in KRB containing 2.8 mM glucose. The KRB was then replaced by KRB containing 2.8 mM glucose for 1 h (basal), followed by additional 1 h incubation in KRB containing 16.7 mM glucose. Islets were extracted with 0.18 N HCI in 70% ethanol for determination of insulin content. Insulin was determined using a human insulin ELISA kit (Alpco, Windham, NH).

### Statistics

Results are presented as means±SE. Statistical calculations were carried out by student's t-test using GraphPad Prism (San Diego, CA). A *p*-value <0.05 was taken to indicated a significant difference.

## Results

### Glucose and fatty acid regulation of UCP-2 protein expression in human islets

**Figure 1:**
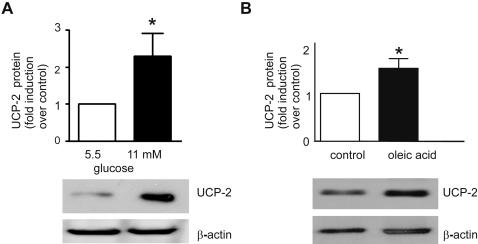
UCP-2 protein is upregulated by glucose and oleic acid in human islets. A Human islets were cultured at 5.5 or 11 mM glucose for 24 hr. B Human islets were cultured at 11 mM glucose plus 1% BSA containing medium (control) or supplemented with 0.45 mM oleic acid for 24 hr. Total protein extracts were analyzed on a Western blot using UCP-2 antibodies. *Upper panel,* The intensities of the protein signal were quantified by scanning of images; *lower panel*, the UCP-2 and β-actin in one representative experiment. All values are mean±SE of at least four independent experiments from separate human islet donors (*t*-test; *, *p*<0.05 relative to control).

UCP-2 protein expression was increased 2.6±0.6 (*p*<0.05) fold in islets cultured for 24 hr at 11 mM glucose, when compared to islets cultured at 5.5 mM glucose (Fig. 1A). Insulin release from human islets increased 4±1-fold in response to 11 mM glucose (*p*<0.05, data not shown). In order to evaluate the effect of free fatty acids on UCP-2 protein expression in the presence of high glucose, isolated human islets were cultured for 24 hr in 11 mM glucose containing medium with or without 0.45 mM oleic acid. As shown in figure 1B, oleic acid potentiated the effect of high glucose by increasing UCP-2 expression 1.5±0.1-fold (*p*<0.05) in human islets in the presence of 11 mM glucose when compared to islets cultured with 11 mM glucose without oleic acid. Insulin release from human islets increased (1.6±0.2-fold, *p*<0.05) in response to oleic acid (data not shown). We also examined the effect of 0.45 mM oleic acid on UCP-2 expression at 4 mM glucose. Under these conditions, oleic acid increased UCP-2 expression by 1.3±0.1-fold (data not shown).

### Effect of dominant negative UCP-2 misexpression on insulin secretion by human islets

**Figure 2:**
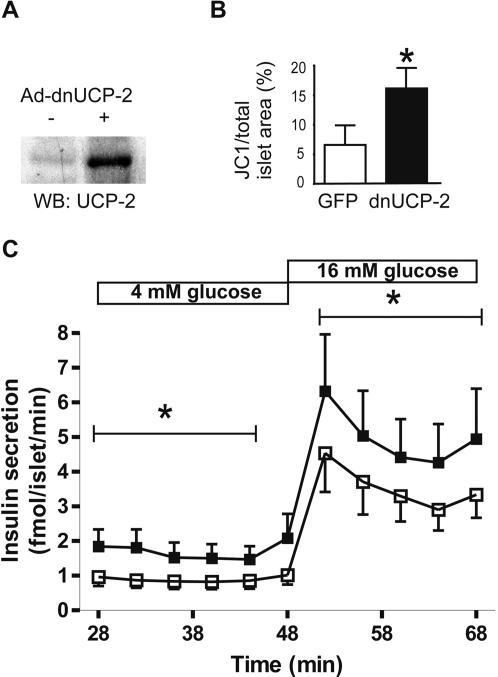
Dominant negative UCP-2 enhances insulin secretion in human islets. A Levels of UCP-2 protein in human islets following adenoviral misexpression of dn-UCP-2 (+) or GFP (−). B DnUCP-2 increases mitochondrial membrane potential. Human islets grown on extracellular-matrix-coated slides were transduced with Ad-dnUCP-2 or Ad-GFP. Mitochondrial membrane potential was detected by live staining with JC-1, and the JC-1 and islet cell area were measured using Image-Pro Plus software. Bar graph shows the percentage of JC-1 (area) divided by total islet area (*t*-test; *, *p*<0.05 relative to control). C Human islets were transduced with Ad-dnUCP-2 (▪) or Ad-GFP (□, control) and cultured in suspension at 5.5 mM glucose before perifusion. Islets were perifused at 4 mM and 16 mM glucose and insulin concentrations were measured every 4 min. Results are of four independent experiments using four separate human islet donors. Data are presented as means±SE and analyzed by *t*-test, *, *p*<0.001.

Adenoviral misexpression of dnUCP-2 resulted in an approximate 4-fold increase in UCP-2 protein expression compared to control human islets (Fig. 2A). Furthermore, adenoviral-mediated increase in UCP-2 protein expression indicates that this band is indeed UCP-2. To evaluate whether dnUCP-2 expression alters mitochondrial membrane potential, dnUCP-2 and GFP expressing human islets were stained with a mitochondria-specific voltage-sensitive JC-1 dye. The intensity of JC-1 red fluorescence is increased as mitochondrial membrane potential is increased [33], [34], [43]. The islets that expressed dnUCP-2 had a significant increase in membrane potential when compared to control islets (Fig. 2B). To determine the potential for UCP-2 to regulate insulin secretion in human islets, they were transduced with Ad-dnUCP-2 or Ad-GFP and perifused (Fig. 2C). The relative increase in insulin secretion in dnUCP-2 expressing islets compared to GFP expressing islets was 85.6±12.7% (*p*<0.001) at basal glucose (4 mM), whereas during glucose stimulation (16 mM), the relative increase was 44.2±8.5% (*p*<0.001) in islets expressing dnUCP-2 vs. GFP.

### UCP-3 expression in human islets

**Figure 3:**
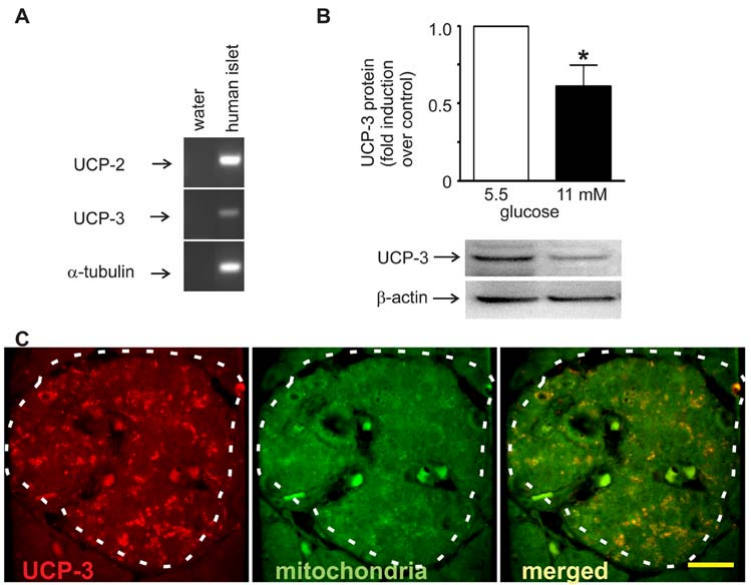
UCP-3 is expressed in human islets. A Human UCP-2, UCP-3 and α-tubulin mRNA expression in human islets. B Human islets were cultured at 5.5 or 11 mM glucose, and total protein extracts were analyzed on a western blot using UCP-3 antibodies. The intensities of the protein signal were quantified by scanning of images; blots for UCP-3 and β-actin is shown for one representative experiment. Data are expressed as means±SE, n = 3, * *p*<0.05. C UCP-3 is expressed in human pancreatic islets, where it colocalizes with mitochondria. A representative layer of human pancreata is depicted showing UCP-3, mitochondria and overlay. Images were acquired using confocal microscope and imaged at x63 magnification, bar = 20 µm.

A single mRNA band of the expected size for UCP-3 was amplified from human islets (Fig. 3A). The cloning and sequencing of the PCR fragment confirmed perfect homology with the published long form of human UCP-3 mRNA sequence (GenBank Accession number: NM003356). Having identified transcription of UCP-3 in human islets, we used quantitative PCR in order to measure the relative abundance of UCP2 and UCP3 mRNA in human islets. Human islets had 8.1±1.4-fold more UCP2 compared to UCP3 mRNA (*p*<0.05, n = 25, different glucose concentrations). We next investigated UCP-3 protein expression in human islets using western blotting and immunohistochemistry. UCP-3 protein expression was decreased by 40±14% in human islets cultured at 11 mM glucose, when compared to islets cultured at 5.5 mM glucose (*p*<0.05, Fig. 3B). To examine the expression of UCP-3 in the pancreas, human pancreatic sections were immunostained with an antibody against the C-terminus of UCP-3. To test antibody specificity, we blocked the UCP-3 antibody signal with specific UCP-3 peptide before staining. No positive staining was observed (data not shown). Next we stained human pancreatic tissue with UCP-3 and mitochondria antibodies (Fig. 3C). The beta-cells had a punctuate staining of UCP-3 that colocalized with mitochondria in beta-cells.

**Figure 4:**
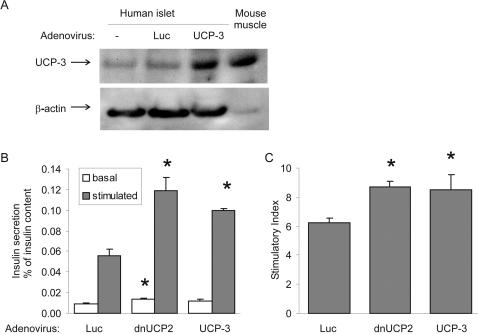
UCP3 over expression in human islets increases glucose-stimulated insulin secretion. A Western blot analysis of UCP-3 expression in transduced islets. B Isolated human pancreatic islets were cultured on extracellular matrix-coated dishes and transduced with adenoviruses expressing control virus (luciferase, Luc), UCP-3 or dnUCP-2. Basal and stimulated insulin secretion indicate the amount secreted during 1-hour incubations at 2.8 (basal) and 16.7 mM (stimulated) glucose following the 2-day culture period after transduction, normalized to whole islet insulin content. C Stimulatory index denotes the amount of stimulated divided by the amount of basal insulin secretion. Data represent results of two different experiments from two different organ donors in quadruplicate. Results are means±SE, *p<0.05 compared to control.

To investigate the effect of UCP-3 overexpression on GSIS, we prepared UCP-3 adenoviruses. Adenoviral overexpression of UCP-3 resulted in an approximate 3-fold increase in UCP-3 protein expression compared to control human islets (Fig. 4A). Forty-eight hr after transduction, we performed GSIS assays in human islets. No significant changes in insulin secretion were observed at basal levels at 2.8 mM glucose in UCP-3 expressing islets compared to control islets. However, as already seen in the perifusion experiment, dnUCP-2 increased basal insulin secretion by 1.5-fold compared to untreated control islets (*p*<0.05). Stimulated insulin secretion at 16.7 mM glucose was significantly increased in human islets expressing UCP-3 or dnUCP-2 (1.8- and 2.1-fold, respectively, p<0.05, Fig. 4B) compared to scrambled control. This resulted in a 37% and 40% increase in the stimulatory index by UCP-3 and dnUCP-2, respectively in human islets (p<0.05, Fig. 4C).

## Discussion

UCP-2 expression is increased in rodent islets in response to either chronic high glucose or free fatty acid concentration exposure and diabetic human islets [17]–[22], [28], [44]. This increased UCP-2 expression may contribute to impaired GSIS in type-2 diabetes [28]. Uncoupling proteins therefore have become an interesting focus for hyperglycemia mediated impaired insulin secretion.

Brown et al. reported that UCP-2 mRNA levels were increased three-fold in human islets cultured at 22 mM glucose for 24 hr [27]. We observed a similar increase in UCP-2 protein when cells were cultured at the presence of 11 mM glucose for 24 hr, glucose concentration typically present in humans with diabetes. In line with these data and the concept of glucotoxicity, UCP-2 overexpression impairs β-cell function [45]. This increased UCP-2 expression in the presence of glucose was further amplified by 50% by addition of the actively oxidized fatty acid oleic acid to human islets at a concentration typically present in diabetes (∼0.45 mM). These data are in agreement with the increase of UCP-2 expression by free fatty acids in isolated rodent islets and in cultured mouse insulinoma cells [18], [20], [44], [46]. This may also be a possible underlying mechanism of free fatty acid induced impaired GSIS in human islets shown during perifusion [47] as well as during static incubation [48]. Taken together these data suggest that the chronically increased glucose and fatty acid concentrations typically present in type-2 diabetes would have an additive effect to increase the expression of UCP-2 protein in islets, an effect that would be expected to attenuate GSIS. The fact that both glucose and oleic acid had this effect implies that the upregulation of UCP-2 protein expression is signaled by traffic through the tricarboxylic acid cycle or a down stream effect of this.

Having established that UCP-2 is upregulated under conditions present in type-2 diabetes (high glucose concentrations and free fatty acid concentrations) we sought to establish if the endogenous UCP-2 protein expressed in human islets plays a role in insulin secretion. Rodent studies demonstrated that islets isolated from UCP-2 knock-out mice, when compared to wild-type islets, secrete more insulin when cultured in basal and high glucose concentrations [22]. Therefore to establish if the endogenous UCP-2 protein expressed in human islets regulated insulin secretion we employed a dominant negative UCP-2 approach in human islets. Using this approach we report increased insulin secretion in response to basal as well as high glucose concentrations in isolated perifused human islets expressing a dominant negative form of UCP-2.

We also report that UCP-3 is expressed in human islets. Previously, when the PCR method was used to amplify UCP RNAs in human islets, only UCP-2 RNA was detected [23]. This first report of UCP-3 expression in human islets poses additional questions about the potential role of UCPs in the modulation of insulin secretion in response to glucose. We investigated the relationship between UCP-2 and UCP-3 mRNA in human islets and report that the level of UCP-3 mRNA expression in human islets was only around 10% of that of UCP-2. We also report that UCP-3 protein is expressed in human pancreatic islets. Chronic exposure of islets to high glucose concentrations attenuated UCP-3 protein expression, whereas UCP-2 protein levels were increased. This suggests a different role of UCP-3 on glucose homeostasis. UCP-3 protein levels were lower in skeletal muscle of prediabetic (impaired glucose tolerance) and type-2 diabetic subjects compared to healthy control subjects [49], [50]. Furthermore, it was reported that UCP-3 protein level was negatively correlated with plasma glucose and insulin levels [49]. Moreover, mice overexpressing UCP-3 have reduced fasting plasma glucose and insulin levels indicating improved glucose homeostasis [49], [51], [52].

Because UCP-3 protein levels were decreased at high glucose concentrations, we over-expressed UCP-3 in isolated human islets and measured insulin secretion at basal and increased glucose concentrations. Interestingly, both UCP-3 overexpression and UCP-2 depletion resulted in a 1.4-fold increased GSIS index in isolated human islets. Recent studies demonstrated that modest overexpression of UCP-3 in muscle cells suppressed reactive oxygen species (ROS) production and increased fatty acid oxidation [40]. These results suggest that increased expression of UCP-3 may be beneficial by preventing harmful ROS production and protecting mitochondria against fat accumulation by improving fatty acid metabolism [15], [40], [53]. Therefore, low UCP-3 levels may contribute to the pathogenesis of type-2 diabetes [54].

In summary, we report here that UCP-2 protein expression is increased in response to glucose in human islets, confirming the corresponding findings made in rodent islets. Moreover, by use of a dominant negative approach we demonstrate that endogenous UCP-2 expression in human islets negatively regulates insulin secretion. We report that UCP-3 is also expressed in human islets, and its expression is decreased in response to high glucose concentrations. Finally, we demonstrate that GSIS is increased human islets over expressing UCP-3. These studies support the emerging concept that UCP-2 (and possibly UCP-3) expression in human islets may contribute to decreased GSIS in patients with type-2 diabetes who are exposed to chronically increased glucose levels. Moreover, these data imply that UCP-2 and UCP-3 may have distinct roles in regulating beta-cell function. Increased expression of UCP-2 in humans with chronic hyperglycemia and/or increased fatty acid concentrations may contribute to impaired GSIS. On the other hand, decreased expression of UCP-3 in high glucose concentrations may impair beta-cell function, implicating that mechanisms that increase UCP-3 expression and/or function are potential therapeutic targets to offset defects of insulin secretion in humans with type-2 diabetes.
