# Correction: UCP-2 and UCP-3 Proteins Are Differentially Regulated in Pancreatic Beta-Cells

**DOI:** 10.1371/journal.pone.0271783

**Published:** 2022-07-14

**Authors:** Yunfeng Li, Kathrin Maedler, Luan Shu, Leena Haataja

After this article [1] was published, concerns were raised about vertical discontinuities suggestive of image splicing in Fig 2A and Fig 3A. Underlying data provided for Fig 2A are in S1 File, and for Fig 3A are in S2 File. While the resolution in S2 File is not sufficient to enable a detailed analysis, the image data support that water controls were included in the experiment, and that UCP2, UCP3, and b-actin PCR reactions each yielded a single visible band. The concern with Fig 2A was not substantiated after investigation.

In discussing this matter, the authors clarified that there is an error in the published Fig 3A, the control panel was erroneously labelled as α-tubulin instead of β-actin. The following primer sequences were used for the β-actin control reactions in this experiment: 5’GTGATGGTGGGCATGGGTCA3’ and 5’TTAATGTCACGCACGATTTCCC3’.

All raw data underlying the results reported in the article are available from the authors as laboratory book copies.

## Supporting information

S1 File Underlying data for the western blot in Fig 2A. (TIF)

S2 File Underlying data for the PCR in Fig 3A. (TIFF)
