# Supplementary figures and images for: Correction: UCP-2 and UCP-3 Proteins Are Differentially Regulated in Pancreatic Beta-Cells
Source: PLoS One. 2022 Jul 14;17(7):e0271783. doi: 10.1371/journal.pone.0271783 (PMC9282598; doi:10.1371/journal.pone.0271783)

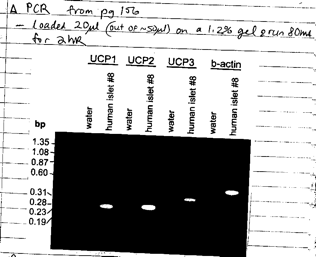

Supplement: S2 File — (TIFF) [file pone.0271783.s002.tiff]
